# Deep-learning-based precise characterization of microwave transistors using fully-automated regression surrogates

**DOI:** 10.1038/s41598-023-28639-4

**Published:** 2023-01-26

**Authors:** Nurullah Calik, Filiz Güneş, Slawomir Koziel, Anna Pietrenko-Dabrowska, Mehmet A. Belen, Peyman Mahouti

**Affiliations:** 1 Department of Biomedical Engineering, Istanbul Medeniyet University, Istanbul, 34220 Turkey; 2 Department of Electronic and Communication Engineering, Yıldız Technical University, Istanbul, 34220 Turkey; 3 Engineering Optimization & Modeling Center, Department of Technology, Reykjavik University, Menntavegur 1, 102 Reykjavik, Iceland; 4 Faculty of Electronics, Telecommunications and Informatics, Gdansk University of Technology, Narutowicza 11/12, 80-233 Gdansk, Poland; 5 Department of Electric and Electronic Engineering, Iskenderun Technical University, Hatay, 31330 Turkey; 6 Department of Avionics, Yıldız Technical University, Istanbul, 34220 Turkey

**Keywords:** Electrical and electronic engineering, Computational science

## Abstract

Accurate models of scattering and noise parameters of transistors are instrumental in facilitating design procedures of microwave devices such as low-noise amplifiers. Yet, data-driven modeling of transistors is a challenging endeavor due to complex relationships between transistor characteristics and its designable parameters, biasing conditions, and frequency. Artificial neural network (ANN)-based methods, including deep learning (DL), have been found suitable for this task by capitalizing on their flexibility and generality. Yet, rendering reliable transistor surrogates is hindered by a number of issues such as the need for finding good match between the input data and the network architecture and hyperparameters (number and sizes of layers, activation functions, data pre-processing methods), possible overtraining, etc. This work proposes a novel methodology, referred to as Fully Adaptive Regression Model (FARM), where all network components and processing functions are automatically determined through Tree Parzen Estimator. Our technique is comprehensively validated using three examples of microwave transistors and demonstrated to offer a competitive edge over the state-of-the-art methods in terms of modeling accuracy and handling the aforementioned issues pertinent to standard ANN-based surrogates.

## Introduction

Low-cost and accurate models of microwave transistors are indispensable in simulation of active circuits. This, in turn, is essential for efficient circuit characterization and design. Design procedures generally require behavioral models of the transistors, which adequately represent their large- and small-signal characteristics over a range of biasing conditions. Standard transistor models for microwave and RF applications usually employ physics-based equations^1^. However, such models come short in certain aspects, e.g., reliable capturing of the electrical characteristics. Furthermore, the development of modelling equations for new physical phenomena requires considerable expertise. Finally, parameter extraction for equation-based models is challenging and difficult to automate^1^. An alternative for equation-based models are lookup table (LUT)-based methods^2,3^. However, these techniques require long SPICE simulation time, as well as suffer from convergence issues for large-scale designs. In addition, LUT-based models lack the control parameters that can be used to manipulate the output characteristics of the model.

Over the recent years, the role of Artificial Intelligence (AI)-based techniques has been continuously growing in the development of efficient numerical procedures for RF and microwave engineering^4^, including data-driven surrogate modeling methods. AI-based modeling has the potential to tackle the limitations conventional approaches mentioned in the previous paragraph^5^. Being data-driven, AI methods eliminate the need for laborious equation development, and engaging the underlying device physics. This results in expediting the model development process and making it more versatile^4^.

One of the AI techniques commonly incorporated for RF device modelling are Artificial Neural Networks (ANNs). ANNs have a history of being used for constructing models of semiconductor devices, especially in RF applications^6–8^. The fundamental advantage of ANN is that it can represent highly nonlinear relations between the system parameters and its outputs without relying on any explicit analytical formulas. ANN model extraction is generally based on the concept of empirical risk minimization; therefore, local minima may be identified instead of a global optimum in some cases^5^. This becomes a challenging problem in modelling of highly nonlinear RF characteristics within broad ranges of input parameters.

*S-*parameters are widely used for RF system characterization. They are easy to measure, and readily convertible to other parameters, and suitable for analysis of both passive and active components^9–11^. Furthermore, *S*-parameters of an active device can also be used for calculation of input and output impedance, isolation, gain, and stability, which are all crucial in the design of small-signal or low-noise amplifiers^11^. At the same time, transistor *S*-parameters are highly dependent on the frequency, biasing conditions, and the temperature. Their accurate rendition is pivotal to reliable design of RF systems.

ANNs have shown a great potential in the design of a variety of RF and microwave components, such as antennas^12^, reflectarrays^13,14^, microstrip filters^6,15^, as well as modelling of *S*- and *N*-parameters of microwave transistors. Their advantage is to construct the model exclusively based on the sampled data from the system, without the necessity to extract an equivalent‐circuit model, or to engage an expert knowledge^4^. Notwithstanding, a rendition of globally accurate surrogates is challenging due to dimensionality issues, the need for representing transistor characteristics over a broad range of parameters and frequency, and sheer handling of highly nonlinear responses^16^. Some of these issues can be alleviated by method such as model order reduction^17^, principal component analysis^18^, high-dimensional model representation^19^, or variable-resolution techniques^20,21^.

An important consideration in constructing highly accurate ANN models is allocation of the training and testing data sets. This includes a sufficient coverage of the input and output space of the model through appropriate sampling strategies, as well as the exclusion of samples from certain regions of the space^4^. For example, in modelling of microwave transistor for LNA designs, a designer might want to exclude training samples pertinent to higher DC currents, where the transistor would not act as an amplifier, or can only use a narrow range of frequency samples from the provided data, corresponding to the target application^22–30^. Such methods can significantly increase the performance of ANN models by reducing the complexity of the dataset, yet, might be detrimental for the versatility of the ANN-based modeling framework. On the other hand, expanding the range of inputs to improve generality would significantly reduce the predictive power of ANN models. The major challenges related to general-purpose surrogate modelling of transistors can be summarized as follows: (i) several responses must be represented (eight, including real and imaginary parts of all *S*-parameters), (ii) the model should be valid over broad ranges of input and output parameters, (iii) the model accuracy should be maintained despite highly-nonlinear relations between the input and the output space.

With the recent developments of high-performance hardware systems, application of Deep Learning (DL) methods^31,32^ has been constantly increasing. DL has been demonstrated to offer improved handling nonlinear system outputs as compared to more traditional regression models (e.g.,^33,34^). Nevertheless, DL techniques face certain problems on their own, especially related to complex model setup (adjustment of hyper-parameters and the network architecture, preventing overtraining, etc.^31,35–37^. Mitigation of these issues can be achieved by means of automated architecture determination through numerical optimization^16,38,39^. Recently, automated architecture determination using Tree Parzen Estimator (TPE) has been reported^40^. In^31^ and^39^, the ANN model components such as the number of layers and hidden neurons, as well as activation functions have been determined using TPE-aided strategy to yield a surrogate model featuring excellent generalization capability and predictive power superior over the state-of-the-art benchmark methods.

In pursuit of realizing further improvements of ANN surrogates, especially in the context of modeling microwave transistors, this work proposes a novel Fully Adaptive Regression Model. Therein, the number of neurons in all layers, the choice of the activation function, the input data pre-processing techniques, as well as the loss functions, are all taken as optimizable parameters, adaptively adjusted using TPE in the course of the model identification. As demonstrated using three examples of microwave transistors, the performance of the FARM surrogates is superior over state-of-the-art modeling methodologies. Design utility of the presented framework is illustrated through application case studies.

The novelty and the technical contribution of this work include: (i) the development of a fully adaptive DL based surrogate for reliable modeling of microwave transistors, (ii) implementation of the framework with automated determination of model architecture and hyperparameters, (iii) demonstration of superior performance of the proposed surrogate in terms of accurate representation of scattering parameters, (iv) demonstration of utility of the model for design of microwave devices (here, Small Signal Amplifiers (SSA)).

## Proposed modeling approach: fully adaptive regression model

Machine Learning (ML) methods, including ANNs, address regression problems by creating surrogate models that represent (data-driven) relationships between the input and output spaces pertinent to the system at hand^39^. Their ability to generalize the data is mainly governed by the model hyperparameters^31^. If the parameters are not selected properly, even advanced ML algorithms may exhibit poor performance, which has been demonstrated for, e.g., ANN architectures^41,42^. In particular, if a model suitable for a given input data is not adequately parameterized, either under-fitting or over-fitting may occur^43^.

In this study, in order to overcome these and other issues, only the *N*_*h*_—the number of layers of the network architecture is determined by the user, whereas other attributes are automatically determined through TPE. Furthermore, the adjustment of the number of layer neurons is supplemented by considering five alternative activation functions, the type of the pre-processing technique to be applied to the input data, as well as the loss function to be used in the back propagation. All of these are decided upon in the course of model identification. The resulting model will be referred to as Fully Adaptive Regression Model (FARM).

### Basics of ANN modelling

A general NN model maps the input parameter vector into the output space by using affine transformations that are controlled by the weighting factors within the layers, as well as nonlinear mappings implemented by the activation functions between the layers. The latter enhance the flexibility of the NN models. Formally, a general NN structure is defined as

1 $$f({\mathbf{x}}) = \sigma ({\mathbf{W}}^{K} \cdots \cdots \sigma ({\mathbf{W}}^{2} \times \sigma ({\mathbf{W}}^{1} \times {\mathbf{x}} + {\mathbf{b}}_{1} ) + {\mathbf{b}}_{2} ) \cdots \cdots + {\mathbf{b}}_{K} )$$

where **x** ∈ ℝ^Di×1^, *f*(**x**) ∈ ℝ^Do×1^ are the input and output vectors, respectively, **W**^k^, **b**_k_ are the weight matrices and bias vector, respectively, whereas σ(⋅) is a non-linear activation function. Thus, NN models map the input vector through a composition of nonlinear mappings, each realized by an individual layer of the network. Using backpropagation^44^, or other training methods (e.g.,^45–47^), the learnable coefficients within the layers are adapted to the data and optimized to solve the regression task at hand, given a network architecture. However, determination of the optimum number of neurons and selection of the activation functions is a non-trivial problem. The main contribution of this work is the development of modelling framework in which all model parameters, including those pertinent to the architecture, are determined automatically, here, by means of TPE.

### FARM: general structure

**Figure 1:**
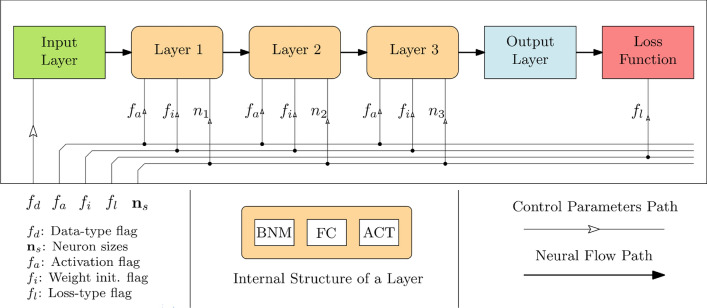
General architecture of the FARM model. The user only defines the number of hidden layers (set to three in the considered example). Subsequently, the optimum setup of parameters, pre-processing type, and the loss function for model training, are all determined by TPE.

Figure 1 shows a general structure of the proposed FARM. The arrows indicate the flow of neural information, as well as the allocation of the control parameters of the blocks. Herein, the basic blocks are the input layer, hidden layers, and the loss function. In conventional ANN structures, as the number of hidden layers increases, a problem of vanishing gradient emerges^48^. In order to overcome this issue, a batch normalization (BN) layer^49^ is used in this study. Consequently, each layer consists of BN, a fully-connected (FC) part^50^, and the activation function (ACT). The BN layer mitigates the internal covariate shift problem^49^, and facilitates the learning process even though the depth of the model increases.

ANN models offer important solutions to regression problems. However, finding the parameters such as the number of neurons in the layers, and the activation function by trial and error requires user expertise. The main motivation of this study is to find the number of neurons in the layers and other parameters of the ANN model in an automatic manner. For this purpose, TPE^51,52^ is employed in this work. TPE is a sequential model-based optimization (SMBO) approach. SMBO methods sequentially construct surrogate models to approximate the performance of the hyperparameter set **S** based on historical measurements.

TPE algorithm attempts to predict the optimum of the loss functions over the surrogate model domain, which is built on the information gathered from the sequential measurements of the input and output points^49,52^. Unlike grid and random search, TPE does not search through the specific points. A new trial point is selected using the previous measurement data to maximize the expectation; subsequently a new point is tried in the loss function. Optimization algorithms that create a surrogate model over sequential points are called SMBO and are widely used in hyperparameter optimization, especially in DNN models. The generated surrogate functions are capable of handling not only continuous variables, but also discrete, categorical and conditional variables^53^. The TPE is a Gaussian Process (GP)-based algorithm with a tree structure that uses two different functions for building a surrogate model over a threshold value^51,54^. In TPE, surrogate model is generated over the defined domain of the optimization problem, which, for this study, contain the hyperparameters of the FARM. Here, the output of the generated surrogate model is the value of the objective function. The aim of GP is to establish a surrogate model to reduce the output response variance at unobserved points within the defined optimization domain. With each iteration, the next hyperparameter point is tried to be estimated over the previously observed points, and as the number of observations increases, the average response of surrogate model starts to converge.

Let ${y}_{i}=f({{\varvec{\theta}}}_{i})$ be a function that is to be minimized. TPE uses previous observation ${\mathcal{D}}_{1:t}=\left\{\left({{\varvec{\theta}}}_{1},{y}_{1}\right),\left({{\varvec{\theta}}}_{2},{y}_{2}\right),\cdot \cdot \cdot \cdot \cdot \cdot ,\left({{\varvec{\theta}}}_{{\varvec{t}}},{y}_{t}\right)\right\}$ to generate a surrogate model. Herein, input (${\varvec{\theta}}$) and output ($y$) are the observation pairs. TPE tries to fit a probabilistic model for $P\left({{\varvec{\theta}}}^{*}|{\mathcal{D}}_{1:t}\right)$ to estimate the next (better) point. After this calculation, a surrogate model is selected using

2 $$P\left({{{\varvec{\theta}}}_{t+1}}|{\mathcal{D}}_{1:t}\right)= \left\{\begin{array}{cc}\mathcal{l}({{\varvec{\theta}}}_{t+1})& y<{y}_{t+1}^{p}\\ g({{\varvec{\theta}}}_{t+1})& y>{y}_{t+1}^{p}\end{array}\right.$$

where ${y}_{t+1}^{p}$ and $y$ are surrogate prediction and loss function evaluation for ${{\varvec{\theta}}}_{t+1}$ parameter set. TPE uses these hierarchical processes to find best estimation point that maximizes the expected improvement^55^. Herein, there are two density functions for the distribution of hyperparameters. One is $\mathcal{l}(\cdot )$, where the value of the loss function is less than the threshold, and the other is $g(\cdot )$, where the value of the objective function is greater than the threshold. Because TPI suggests better candidate hyperparameters for evaluation, the value in the loss function recovers much faster than random or grid search, which leads to a lower overall evaluation of the loss function. Although the algorithm spends more time choosing the next hyperparameters to improve the model performance, it stands as a more suitable solution in terms of the total time spent on grid search and random search. Hence minimizing the human factor in model design by out the person out of the loop^51^.

The model is subsequently used to choose a new set of hyperparameters. TPE models *P*(***L***_*avg*_|**S**) and* P*(**S**), where **S** represents the hyperparameters, whereas ***L***_*avg*_ is the associated average loss value. In this work Hyperopt^52^ Python package was used for this procedure.

### FARM: data pre-processing type selection

In the realm of regression models, the distribution of the input data is an important consideration as it affects the quality of fitting the model to the output values. Depending on the setup, the input data can be fed to the model either in a raw form, or with a zero mean upon suitable pre-processing. If the ranges of the input parameters differ considerably, ensuring satisfactory model generalization may be problematic. Hence, the data is usually normalized by using *z*-score^56^, or mapping it to the [– 1, 1] range. Either of these procedures directly affects the data distribution. The ultimate goal of these manipulations is to put the data into a format that the model can handle. In this work, the following commonly used four types of input data formats are employed: raw, zero-mean, *z*-score, and min–max normalization. The data-type flag is defined denoted as *f*_*d*_, and can assume a value from the set {0, 1, 2, 3}, according to the list above. This value is to be determined in the course of model training using TPE. Below, a brief characterization of the four data formats has been provided. The purpose is that for any given dataset, it is not known which preprocessing would be appropriate for that data. TPE undertakes the task of overcoming this problem.

We assume that the input data is stored in *N*_*s*_ × *N*_*d*_ matrix ***X***_*rw*_, where *N*_*d*_ is the input space dimensionality, and *N*_*s*_ is the number of training samples. The *raw* format means that the matrix ***X***_*rw*_ is submitted to the model without any processing. In *zero-mean* format, the data is transformed as ***X***_*zc*_ = ***X***_*rw*_ – ***μ***_*x*_, where ***μ***_*x*_ is the column-wise mean of ***X***_*rw*_. ***X***_*zc*_ matrix has the same size as ***X***_*rw*_, and contains data whose average is zero on a columnwise basis. The *z-score* format is defined as

3 $${\varvec{X}}_{zs} = \left[ {{\varvec{X}}_{rw} - {\varvec{\mu}}_{x} } \right]/{\varvec{\sigma}}_{x}$$

where ***μ***_*x*_ and ***σ***_*x*_ are column-wise mean and standard deviation vectors of ***X***_*rw*_. The operators in (3) are understood component-wise. It should be noted that Eq. (3) corresponds to the so-called whitening ^57,58^, where the data is transformed to exhibit zero mean and unity standard deviation. The last format is *min–max normalization*, defined as

4 $$\,\,{\varvec{Z}}_{mm} = \frac{{{\varvec{X}}_{zc} }}{{\max (|{\varvec{X}}_{zc} |)}}$$

Herein, max(|**X**_**zc**_|) defines selection of column-vise maximum element. The min max normalization aims at bringing all values in the zero-centered matrix between [− 1, 1]. For this reason, after taking the absolute value of ***X***_*zc*_ in (4), the column-vise is divided by whatever the maximum element is. All of the mentioned data normalization techniques can be used in the proposed FARM model. The normalization method is determined through TPE, depending on the composition of the available training data. On the other hand, other techniques can be incorporated into FARM as well; however, this would increase the number of training iterations required, without much of additional benefits in terms of the model performance. Consequently, in this study, only raw, zero-mean, z-score, min–max data formats have been taken into consideration as data pre-processing methods. All three preprocessing techniques are applied to the data and each resulting matrix is kept separately. Thus, there are a total of four matrices containing the raw data. TPE tries these matrices and decides which normalization method is more suitable.

### FARM: architecture-related parameters

The proposed FARM surrogate employs the following architectural and ANN-mapping-related parameters, jointly referred to as hyperparameters:

- The number *θ* of neurons in the fully-connected parts of the respective layers. This is one of the most important parameters determining the behaviour of the model. In this work, *θ* can assume values from a discrete set {32, 64, 96, …, 1024}. The vector ***n***_*s*_ ∈ *R*^*Nh*×1^ gathers the *θ*-values for all *N*_*h*_ hidden layers.
- Activation function, which determines input–output behaviour of the neurons. In this work, a discrete set of five functions is considered, as indicated in Table 1. Most of these are static, whereas the Parameterized ReLU function contains a learnable parameter *α*. Selection of activation function is controlled via *f*_*a*_.
- Weight initialization function, which is an important parameter for DNN models. To ensure efficient network learning, randomly assigned values must remain in the operable region of the activation functions, which is controlled by *f*_*i*_. In this work, three different weight initialization function, Xiavier Normal^59^, Orthogonal^60^, and Kaiming Normal^61^, are considered as possible choices for *f*_*i*_.

**Table 1:** Activation functions used by FARM surrogate.

${f}_{a}$	Activation function	Definition
0	Tanh (x)	${\varvec{2}} \times \left( {\frac{1}{{1 + e^{ - x} }}} \right) - 1$
1	ReLU (x)	Max (*x*,0)
2	Parameterized ReLU (x)	Max (*x*,0) + α × min (*x*,0)
3	Leaky ReLU (x) (0.1)	Max (*x*,0) + 0.1 × min (*x*,0)
4	Leaky ReLU (x) (0.01)	Max (*x*,0) + 0.01 × min (*x*,0)

Altogether, the ensemble of parameters (***n***_***s***_, *f*_*a*_, *f*_*i*_) is to be determined during the model training process, which—as mentioned before—is handled by TPE.

Here, it is worth mentioning that some of the parameters such as ${f}_{a}$ and ${f}_{d}$ are categorical, these definitions are pertinent to names but for handing of TPE these names are represented in integer forms (for example: data type flag ${f}_{d}\in \left\{\mathrm{0,1},\mathrm{2,3}\right\}$ and activation function flag ${f}_{a}\in \{\mathrm{0,1},\mathrm{2,3},4\}$), each name associated with a given integer value. The TPE algorithm works with these values and attempts to predict the next set of parameters based on these integers for the mentioned categorical variables.

### FARM: loss function selection

While selecting the architecture of the model, only the parameters related to the hidden layers are sufficient. However, the loss functions used during the training, together with the pre-processing of the data, also significantly affect the training process. Let ***o***_*k*_, ***t***_*k*_ and ***e***_*k*_ be output, target and error vectors of *k*th sample of data set. The model error is then defined as ***e***_*k*_ = ***o***_*k*_* −* ***t***_*k*_. Possible loss functions utilized in this work have been listed in Table 2. The choice of the loss function can significantly affect the training and the performance of a deep neural network^62^. One of the conceptual differences between the proposed work and other studies is that the type of the loss function to be employed within the model is decided upon by TPE with respect to the composition of the data.

**Table 2:** Loss functions employed by FARM surrogate.

${f}_{l} \mathrm{Flag}$	Loss function	Definition
0	Mean square error (MSE)	$\frac{1}{{N_{s} }}\sum\limits_{k = 1}^{{N_{s} }} {\left| {e_{k} } \right|^{2} }$
1	Mean absolute error (MAE)	$\frac{1}{{N_{s} }}\sum\limits_{k = 1}^{{N_{s} }} {\left| {e_{k} } \right|}$
2	Huber loss (***β***)	$$\left\{ \begin{gathered} \frac{1}{{N_{s} }}\sum\limits_{k = 1}^{{N_{s} }} {\left| {e_{k} } \right|^{2} } \,\,\,\,\,\,\,\,\,\,\,\,\,\,\left| {e_{k} } \right| < \beta \hfill \\ \beta \times \left( {\left| {e_{k} } \right| - \frac{1}{2}\beta } \right)\,\,\,\,\,otherwise \hfill \\ \end{gathered} \right.$$

### FARM: model training and validation

**Figure 2:**
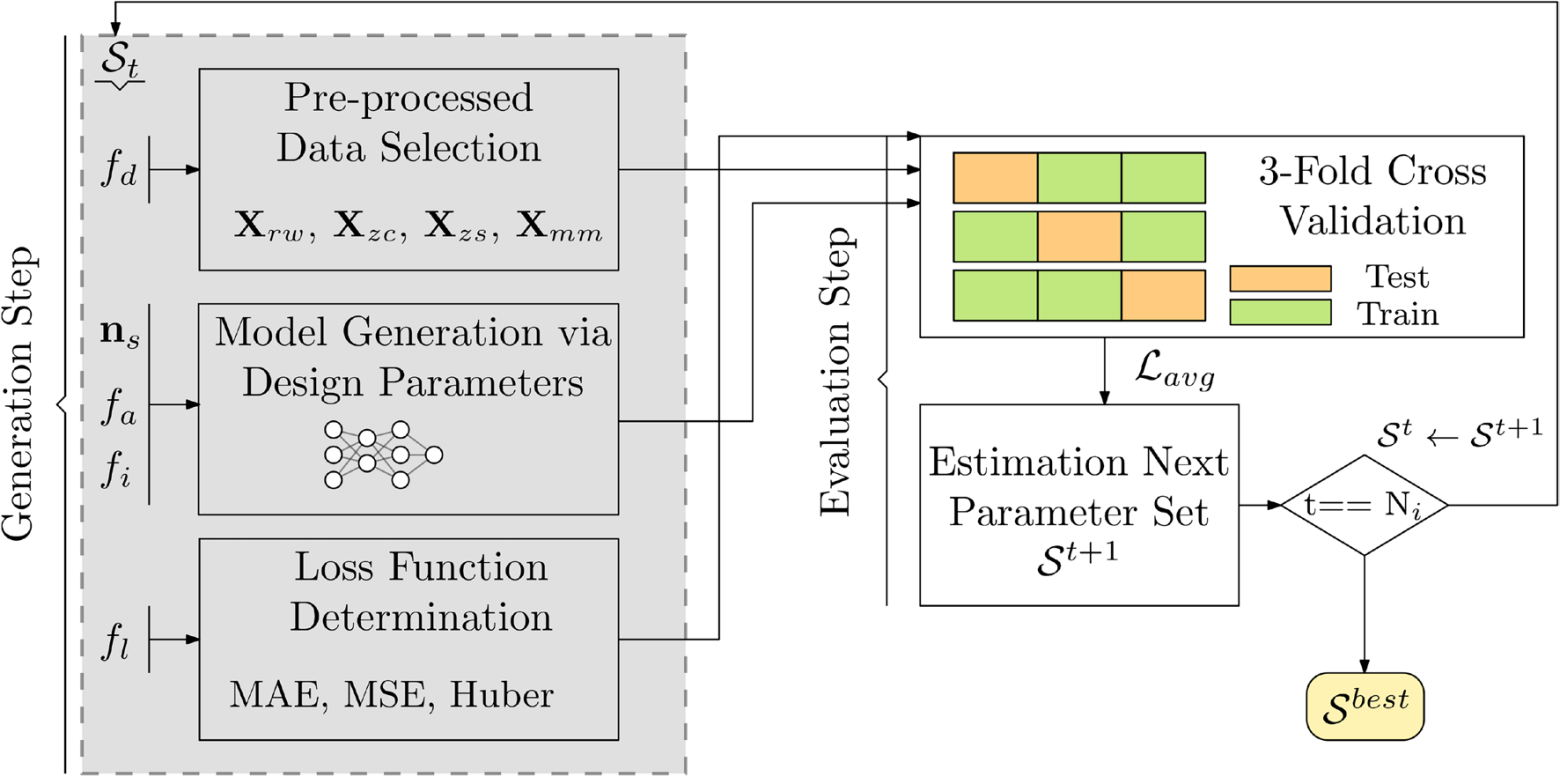
Training of TPE-based FARM model. First, according to the **S**^*t*^ set data type, model architecture and loss function are determined in the Generation Step. Afterwards, TPE is executed using threefold cross validation MAE average in the Evaluation Step. Based on the evaluation, the new parameter set **S**^*t*+1^ is calculated. The best configuration obtained after *N*_*i*_ iterations is selected to be the final parameter setup **S**^*best*^. In this study, *N*_*i*_ = 50. The overall procedure illustrated in the figure is referred to as the *FARM Searching Process*.

The training process consists of the two main stages: *Generation Step* and *Evaluation Step*. After the user has determined the number *N*_*h*_ of hidden layers, the procedure illustrated in Fig. 2 is executed.

The first operation in the *Generation Step* is shuffling the training data randomly according to a uniform distribution. This is necessary as if the data collected sequentially via a certain physical value is used in the training process without mixing, a suitable statistical model of the data space cannot be established during cross validation. In the second step, the matrices ***X***_*rw*_, ***X***_*zc*_, ***X***_*zs,*_, ***X***_mm_, related to the *f*_*d*_ flag are created. The model architecture is determined using the TPE framework, which identifies the parameter set **S** consisting of (*f*_*d*_, **n**_*s*_, *f*_*a*_, *f*_*i*_, *f*_*l*_) discussed earlier, along with the neuron weights, which, altogether, results in the lowest possible average MAE as computed using cross-validation.

In the *Evaluation Step*, the model error (here, MAE) is estimated using threefold cross-validation^63,64^. The estimation is utilized by TPE to generate a potentially more suitable set of model parameters. The model training does not involve the testing data, separated before for the purpose of model validation. The final parameter values **S**^*best*^ are assigned to be the best set found during *N*_*i*_ iterations of the TPE process.

**Figure 3:**
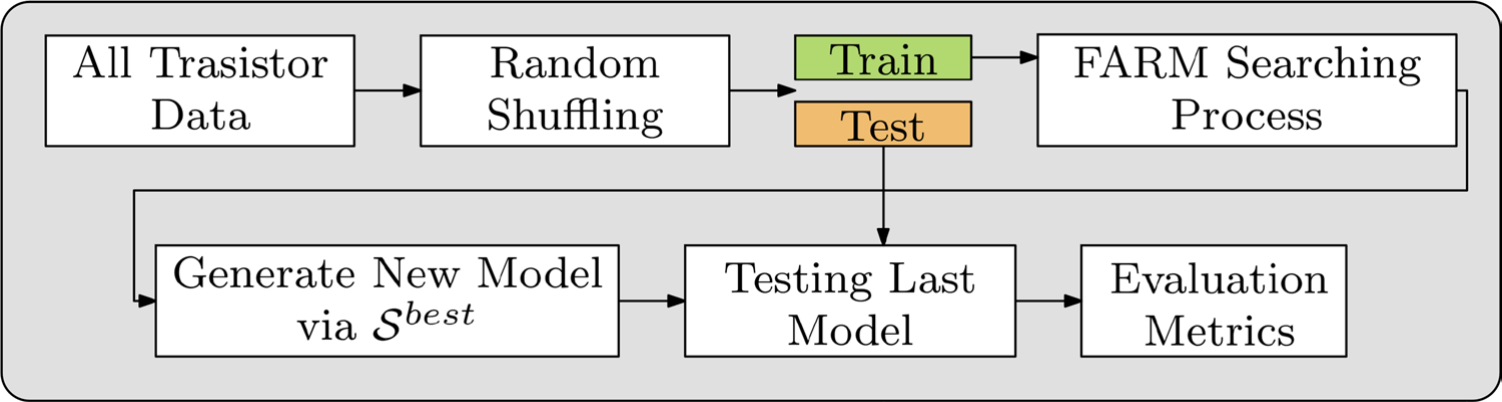
General flow of FARM surrogate construction and validation. Randomly shuffled data is split into training and testing sets. *FARM Searching Process* (cf. Fig. 2) is executed using the training data. The optimum model parameters are determined, and a new model is generated over the best found parameter set **S**^*best*^. Subsequently, the model performance metrics are evaluated using the testing data.

The overall flow of the surrogate model construction and validation has been shown in Fig. 3. As mentioned earlier, the available data is shuffled to ensure that both the training and testing sets—established in the next step—provide sufficiently uniform coverage of the parameter space (which may not be the case without the shuffling: normally, the measurement data is arranged with respect to a specific transistor parameter, e.g., bias voltage). Subsequently, the FARM *Searching Process* is executed on the training data (cf. Fig. 2). The final model is generated using the parameter set **S**^*best*^ identified using TPE using the entire available dataset. The performance metrics are finally evaluated to verify the model quality.

### Verification case studies

**Figure 4:**
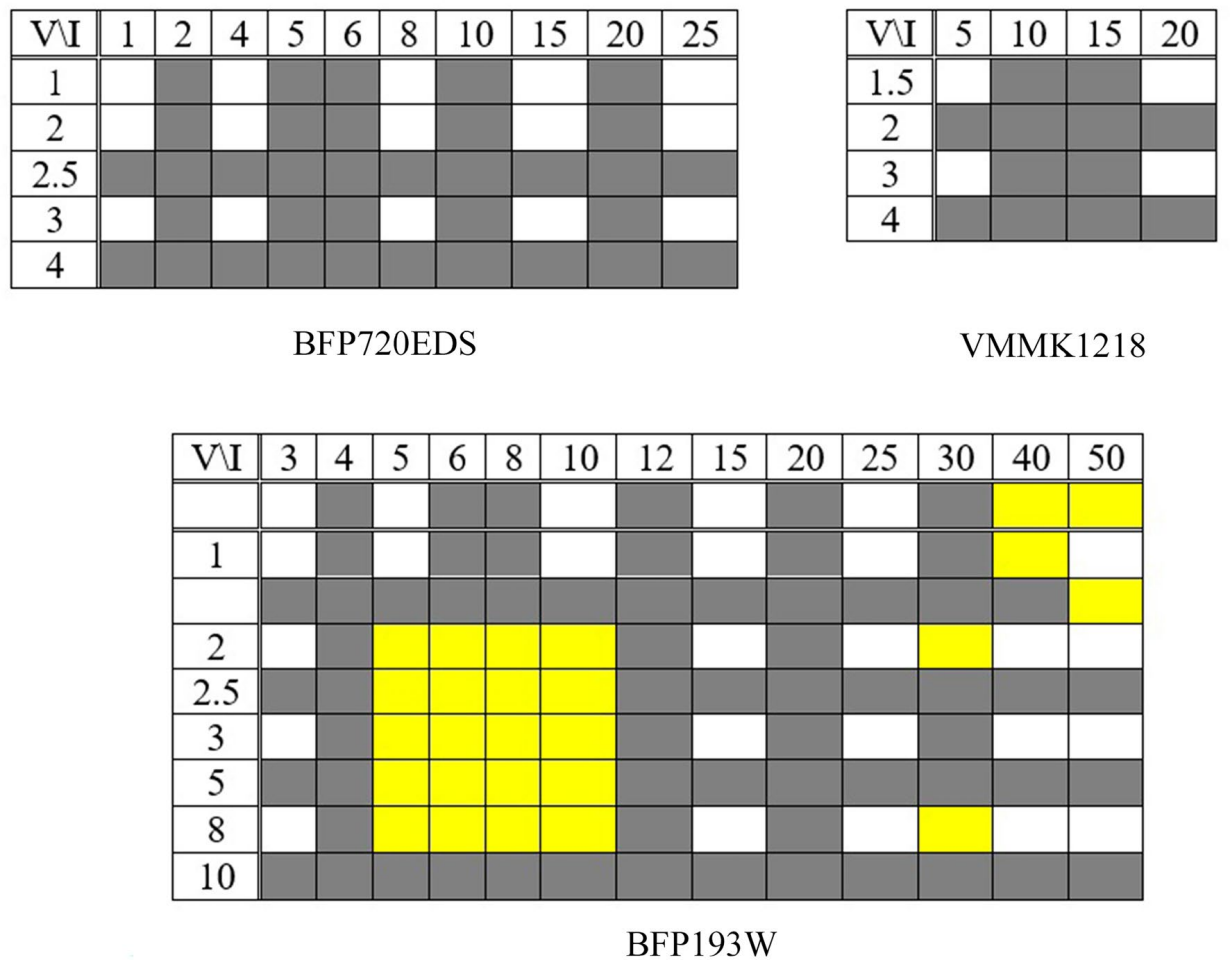
The assignment of training (while boxes) and testing samples (grey boxes) in terms of the bias conditions (V/I). The yellow boxes represent those combinations of bias conditions for which measurement data was not available.

In this work, to verify the proposed model, two different approaches based on experimental data are taken into consideration: (I) investigation of the accuracy of representing the measured scattering parameters of the considered test transistors using the FARM surrogate, (II) an application case study involving design and realization of a small signal amplifier based on the proposed FARM surrogate model. With respect to the first verification approach, the measured scattering parameters of three different transistors, BFP193W (0.01–6 GHz) a NPN silicon, BFP720ESD (0.1–10 GHz) is a silicon germanium carbon (SiGe:C) NPN hetero junction wideband bipolar RF, and VMMK-1218 (0.5–18 GHz) Low Noise E-PHEMT. The measured scattering parameter characteristics of the transistors were acquired from the touchstone files provided by the manufacturer^65–67^, and split into the training and testing set of as it shown in Fig. 4. The frequency ranges of each transistor are defined by the manufacturers, in which the transistors are considered stable and can be used for the design purposes. Going out of these boundaries would be in conflict with the manufacturer’s suggestions and the nature of the transistors with respect to their aimed applications as defined in the datasheets. Furthermore, to facilitate further research by potential readers, the data sets used in this work have been shared in the IEEE data port^68^. Here, the assignment of the training data is chosen to achieve a globally accurate model while using a possibly small number of samples. Furthermore, we also intend to verify the extrapolation capability of the surrogate, therefore, the training points do not cover the entire range of bias voltages.

It should be noted that not only the ranges of DC voltage and current are different for both considered transistors, but the devices also differ in terms of the scattering parameter characteristics. Although *S*_11_ and *S*_22_ have similar ranges for both real and imaginary parts, which is approximately [− 1, 1], the variability range of *S*_12_ is [− 0.15, 0.15], and it is typically is around zero, whereas the range of *S*_21_ is much broader, i.e., [− 70, 70]. Each transistor has three inputs, bias voltage *V*, bias current *I*, and frequency *f*_*r*_. For transistor BFP193W, we have 210 frequency points in the range 0.01 GHz to 6 GHz, whereas for BFP720ESD, we have 233 samples between 0.1 GHz and 10 GHz, and VMMK-1218 have 65 samples between 2 and 18 GHz. In he shared data sets^68^, the 1st, 2nd, and 3rd column contain the input parameters, i.e., DC bias conditions and frequency in GHz, respectively, whereas the scattering parameters of the transistor (*S*_11_, *S*_21_ *S*_12_, *S*_22_) are presented in a rectangular form (real and imaginary part), from the 4th to 11th column, respectively.

At the end of the FARM searching process, the model performance is evaluated using the testing set. The performance of the proposed model has been compared to the state-of-the-art methods. To ensure fair comparison, a Bayesian based hyper-parameter optimization process is applied to each of the benchmark models in order to conduct a comparison based on their optimum performance. The software and hardware setup of the simulation stations are as follows. The platforms used for coding of surrogate modelling algorithms are Pytorch^69^, Hyperopt^52^, and MATLAB. The hardware setup of the used system is AMD Ryzen 7 3700X 8-core 3.59 GHz processor with 32 GB RAM, along with GTX 2080TI on a 64-bit operating system.

### FARM surrogate performance and benchmarking

Table 3 provides a comparison of predictive power of the proposed FARM surrogate and the benchmark models. The presented error values (Mean Absolute Error, ^MEA^^70^) are obtained as a mean of five different model setup runs initialized with different Random Number Generator seeds. The benchmark set includes Support Vector Regression Machine SVRM^71^, and Gaussian Process Regression GPR^72^). Their hyper parameters are optimized using a Bayesian Optimization tool a built-in Matlab algorithm^73^. The user defined parameters of the optimization process were selected as follows: *K*-fold validation with *K* = 3, maximum iteration number of 30, all eligible hyper-parameters of the models are included into the search domain^74,75^. Although both SVRM and GPR surrogates have been demonstrated successful in *S*-parameter modelling^4,6–8,26^, the results obtained in this work suggest the opposite work. The main reason is that applications of SVRM, as presented in the literature, handled problems which are usually limited in terms of the narrow-range input space. For example, the DC current range is typically taken as 5–15 mA or 1–10 mA^26^, where the variability of the scattering parameters is quite limited. In addition to narrow input parameter ranges, the SVRM are typically trained using considerably larger datasets, whereas, in this work, not only a very small portion of the entire data set was taken as the training data, but also a small portion thereof is used in shuffled K-fold validation. These factors make it much more difficult for the algorithm to create an accurate mapping between the inputs and outputs of the model, which is an essential matter for sparse data set regression problems. In order to clearly present this phenomena, the authors added an additional simulation results where the performance of SVRM for narrow-range data sets is studied.

1. For BPF720ESD, the voltage values of [1, 2.5, 4] and [2, 3] volts are taken as training and test sample points respectively for [5, 10, 15] mA sample points.
2. For BFP193W, the voltage values of [0.5, 1.5] and [1] volts are taken as training and test sample points respectively for [5, 10, 15] mA sample points.

**Table 3:** Modeling results and benchmarking.

Transistor	Models		*S*_11_	*S*_21_	*S*_12_	*S*_22_	Average [MAE]
BFP193W	GPR		0.56	6.7	0.037	0.310	1.9
	SVM		0.32	6.5	0.020	0.195	1.77
	This work	FARM single layer	0.08	0.10	0.003	0.028	0.06
		FARM two layers	0.06	0.11	0.010	0.02	0.05
BFP720EDS	GPR		0.46	10.3	0.010	0.41	2.8
	SVM		0.43	8.0	0.018	0.26	2.1
	This work	FARM single layer	0.026	0.456	0.007	0.022	0.13
		FARM two layers	0.025	0.270	0.005	0.021	0.08
VMMK-1218	GPR		0.46	8.2	0.02	0.05	2.18
	SVM		0.64	3.1	0.04	0.45	1.06
	This work	FARM single layer	0.09	0.25	0.03	0.09	0.12
		FARM two layers	0.08	0.23	0.01	0.07	0.10

It should be emphasized that although these selected cases correspond to narrower range in terms of the current domain, still the training and test/hold-out ratio is 66–33%. Under the same training and optimization process (K-fold validation with *K* = 3, Bayesian optimization with maximum iteration count of 30), the performance of the SVRM is significantly improved in this new case study, where the MAE values are reduced almost 10 times, while the performance of the proposed FARM model did not change. For BFP193W-SVRM [old Avg. MAE 1.77 → new Avg. MAE 0.18]. For BFP720EDS-SVRM [old Avg. MAE 2.1 → new Avg. MAE 0.25]. For BFP193W-FARM-Two-Layers [old Avg. MAE 0.05 → new Avg. MAE 0.05]. For BFP720EDS-FARM-Two-Layers [old Avg. MAE 0.08 → new Avg. MAE 0.07]. This demonstrates that SVRM or other benchmark methods are simply unsuitable to handle the complexity of the considered modeling task defined over a large input space and small training/testing datasets.

**Figure 5:**
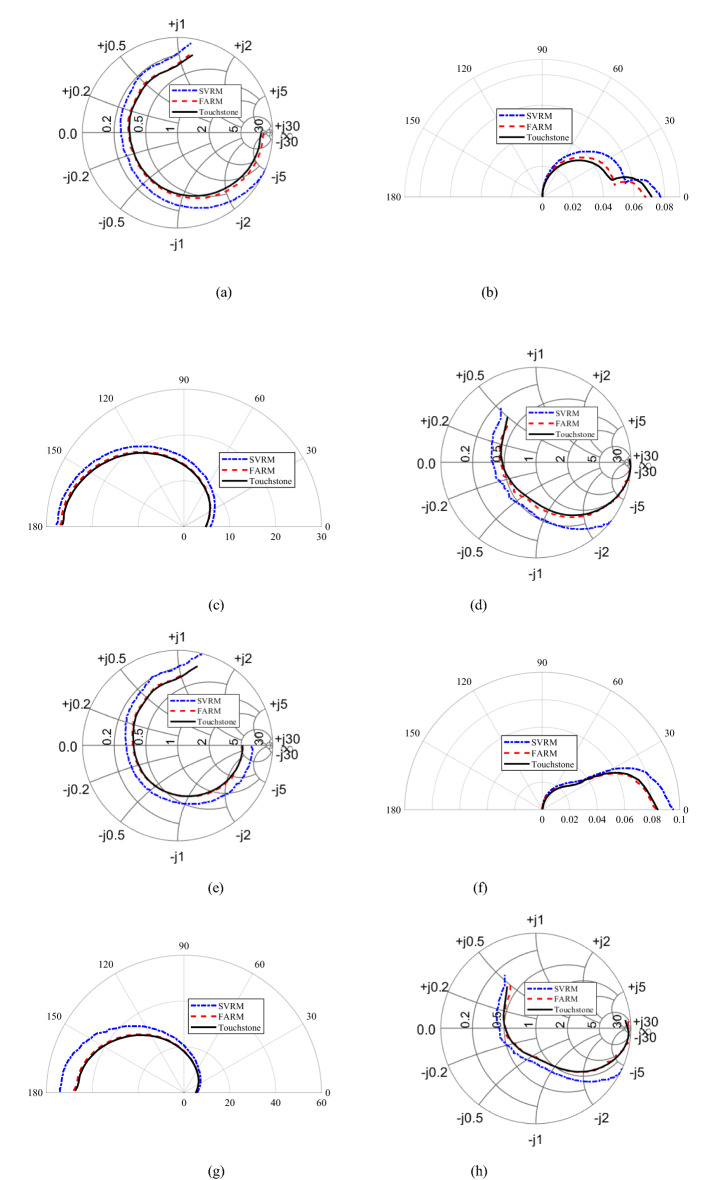
Transistor BFP 720ESD: Smith chart (for reflection coefficient) and polar graphs (for transmission coefficient) of (**a**–**d**) *S*_11_, *S*_12_ *S*_21_, *S*_22_ at bias voltage *V* = 2.5 V and bias current *I* = 10 mA, (**e**–**h**) *S*_11_, *S*_12_ *S*_21_, *S*_22_ at *V* = 4 V and *I* = 25 mA.

**Figure 6:**
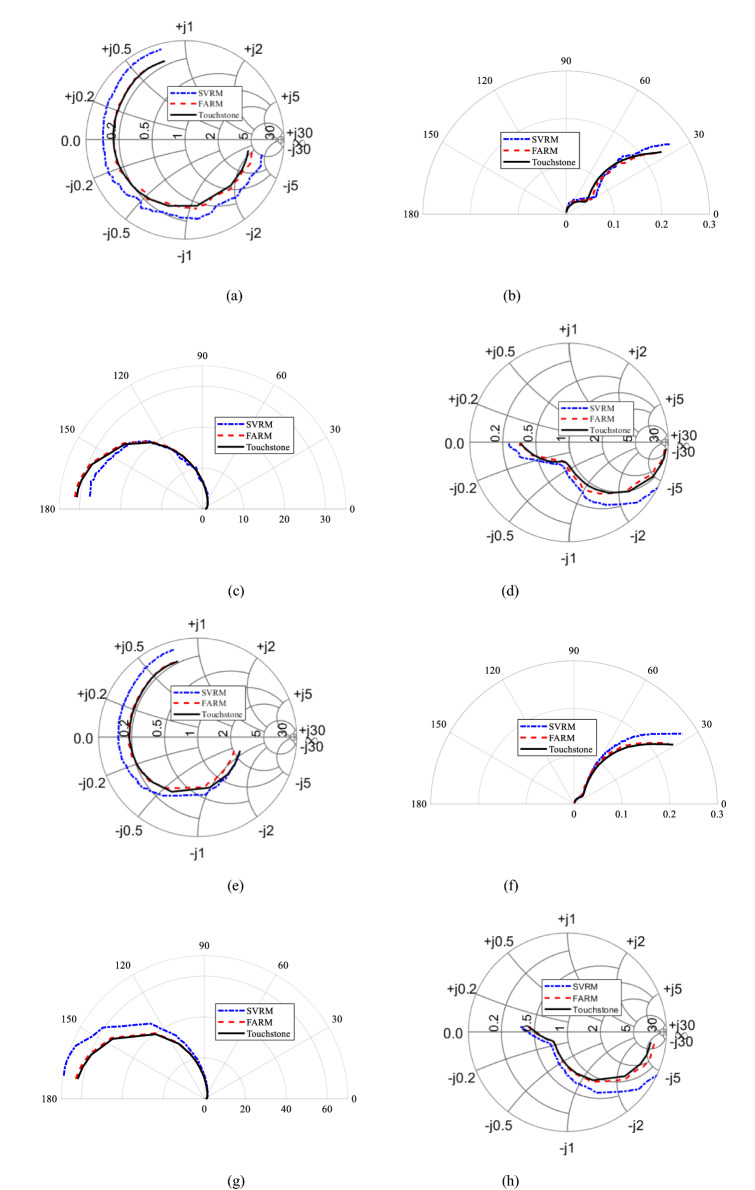
Transistor BFP 193W: Smith chart (for reflection coefficient) and polar graphs (for transmission coefficient) of (**a**–**d**) *S*_11_, *S*_12_ *S*_21_, *S*_22_ at bias voltage *V* = 2.5 V and bias current *I* = 12 mA, (**e**–**h**) *S*_11_, *S*_12_ *S*_21_, *S*_22_ at *V* = 10 V and *I* = 50 mA.

**Figure 7:**
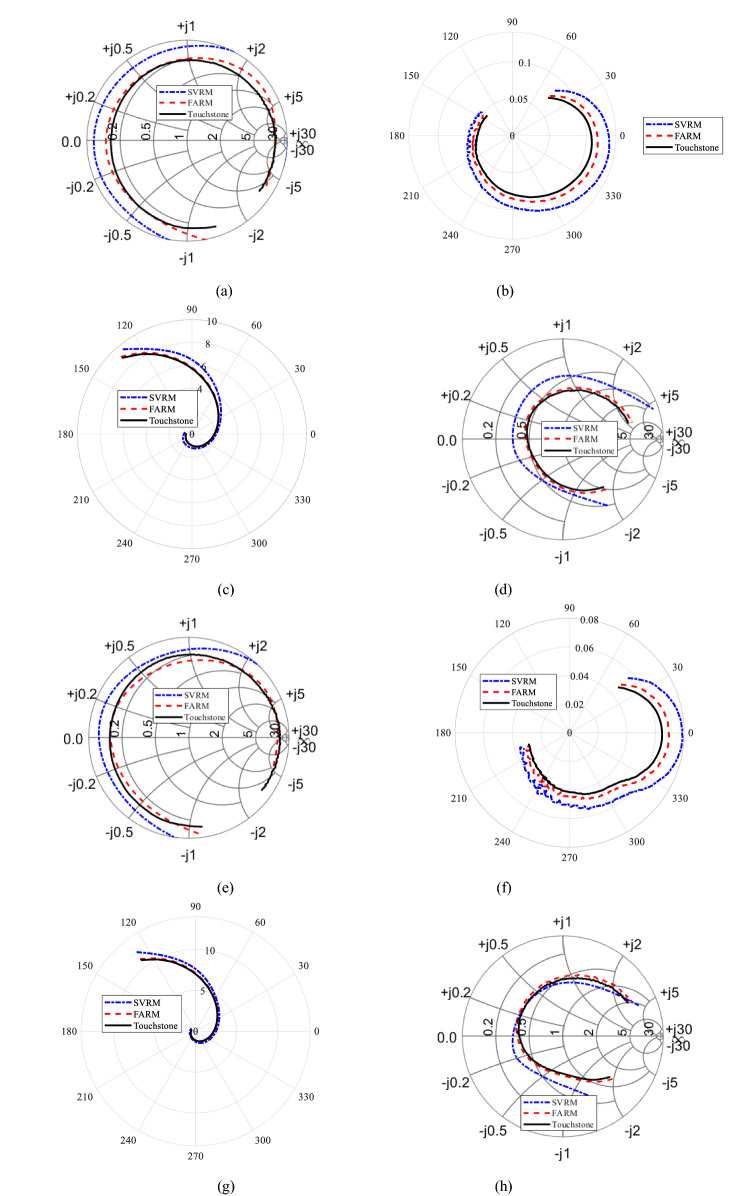
Transistor VMMK-1218: Smith chart (for reflection coefficient) and polar graphs (for transmission coefficient) of (**a**–**d**) *S*_11_, *S*_12_ *S*_21_, *S*_22_ at bias voltage *V* = 2 V and bias current *I* = 10 mA, (**e**–**h**) *S*_11_, *S*_12_ *S*_21_, *S*_22_ at *V* = 10 V and *I* = 50 mA.

Visual comparison of the FARM and SVRM model and their alignment with the measurement data has been shown in Figs. 5, 6 and 7 for selected bias conditions. It can be observed that the visual agreement between the measurement and FARM-predicted characteristics is excellent, as opposed to the SVRM model. Note that some of the presented plots correspond to data extrapolation.

As for the training time of the proposed method, for BFP193W, TPE spends 40–45 min for 50 iterations to estimate the parameters of a single-layer model. The values for BFP720EDS and VMMK-1218, are in the range 30–32 and 14–18 min, respectively. The reason for these variation is that TPE in some cases chooses models with a high number of neurons. Therefore, the computational complexity increases. In some cases, models with smaller number of neurons are established. For two-layer models, the training time ranges from 90 to 96 min for BFP193W, 61 to 65 min for BFP720EDS, and 27–31 min for VMMK-1218. As the number of layers in the model increases, the training time also increases. It should be noted that the user only decides upon the number of layers of the models. All other design parameters are estimated by the TPE algorithm over 50 iterations. This can be reduced with high-end hardware setups (Graphical Processing Unit, GPU) enabling parallel processing. The mentioned results are obtained using the following setup: AMD Ryzen 7 3700X 8-Core Processor 3.59 GHz, with 32.0 GB of installed RAM, and Nvidia 2080 GPU 8 GB.

### Application case study: FARM surrogate for SSA design

**Figure 8:**
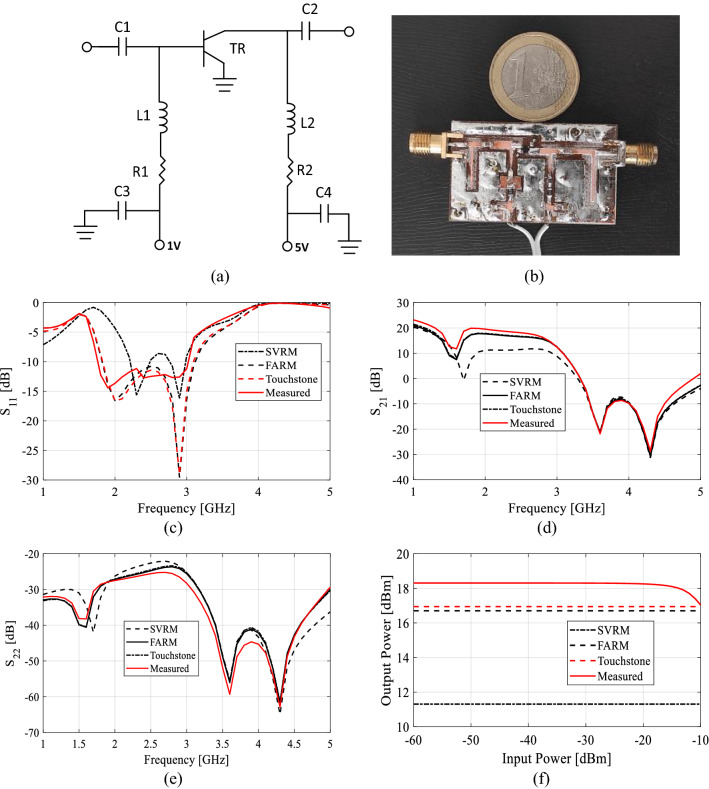
Low-noise amplifier designed using the proposed FARM surrogate: (**a**) schematic diagram of the SSA, (**b**) photograph of the circuit prototype (the SSA designed using the FARM model with two internal layers), comparison of simulated results from Touchstone, FARM and SVRM model with measured (**c**) S_11_, (**d**) S_21_, (**e**) S_12_, (**f**) Pout @ 2.4 GHz, characteristics of SSA.

As for a secondary verification of reliability of the proposed method, design and realization of a small signal amplifier is presented in this section. To demonstrate design utility of the proposed FARM surrogate, the obtained small signal model of BFP720EDS is employed to design a Small Signal Amplifier (SSA). The SSA schematic is presented in Fig. 8a. Therein, the matching network architecture and the component values are established using the manufacturer touchstone data.

The circuit has been implemented on FR4 substrate. The operating frequency is 2.4 GHz, whereas the biasing voltage and current are 2.5 V and 10 mA, respectively. Figure 8b shows the photograph of the SSA prototype. Here in order to clearly present the effect of miss-prediction of scattering parameters on the actual performance of a SSA design, instead of touchstone values, scattering parameters obtained from SVRM and FARM are applied to the circuit design with touchstone files. The simulated SVRM, FARM, touchstone, and the measured S-parameters of the circuit can be found in Fig. 8c–e and *P*_*out*_ in Fig. 8f. However, it should be mentioned that the predictions of surrogate models are inferior beyond – 15 dBm input power due to the lack of small-signal model capabilities to predict the 1 dB compression point. In order to be able to calculate parameters such as OIP3, *P*_*out*_, etc., instead of the small-signal model (models that corresponds to one bias condition), which is used in this work, a large-signal model which is capable of predicting nonlinearity of the output power should be used instead. Table 4 provides the optimal values of FARM models while Table 5 presents the design parameters of SSA. Note a good agreement between FARM-based predictions, touchstone and the measurements. At the same time, the predictions based on the SVRM are significantly worse.

**Table 4:** Optimally selected farm parameters.

Transistor	Layer size	FARM properties				
		*N*_*a*_	Loss function	Pre-processing	Weight Initializer	Activation function
BFP193W	Single	864	MSE	Zero Mean	Xavier	PReLU
	Two	288 544	Huber (0.01)	Zscore	Kaiming	LeakReLU (0.01)
BFP720EDS	Single	864	Huber (0.01)	Zscore	Kaiming	ReLU
	Two	736 416	MSE	Min–Max	Xavier	LeakReLU (0.01)
VMMK-1218	Single	864	Huber (0.01)	None	Kaiming	LeakReLU (0.01)
	Two	928 672	MSE	Zscore	Xavier	LeakReLU (0.01)

**Table 5:** Design parameters of the SSA.

C1	100 pF	C2	100 pF
C3	330 nF	C4	330 nF
L1	15 nH	L2	4.7 nH
R1	4.7 KΩ	R2	270 Ω

## Conclusion

This paper introduced a novel approach to data-driven modelling of microwave transistors. The presented methodology, referred to as FARM combines DL techniques with automated determination of the model architecture using Bayesian optimization. It allows for reliable modelling of transistor *S*-parameters over broad ranges of bias conditions and operating frequencies.

Its most important advantage is competitive predictive power as well as extrapolation capability achieved using small numbers of training samples. The latter is practically important due to high cost and technological intricacies related to the acquisition of the measurement data. The aforementioned benefits have been demonstrated using two transistors modelled over wide ranges of bias voltages and currents, as well as comparisons with state-of-the-art surrogates, specifically SVRM and GPR. The obtained MEA errors are as low as 0.05 and 0.08 for the first and the second transistor, respectively. These numbers correspond to excellent visual agreement between the FARM surrogate outputs and measurement data. At the same time, the benchmark techniques exhibit poor performance: despite their proven efficacy, both SVRM and GPR fail under challenging scenarios considered in this work. The design utility of the FARM model has been corroborated through the design of the low-noise amplifier, with satisfactory agreement between the SSA characteristics predicted using our surrogate and the measurements of the circuit prototype. Based on the presented results, the proposed model can be perceived a viable alternative to existing approaches in terms of design-ready behavioural modelling of microwave transistors, especially for applications that require reliable prediction of device characteristics over broad ranges of operating conditions. The future work will include the extension of the proposed work with physical parameter-based data-driven modeling of small signal parameters of a metal–semiconductor field-effect transistor.

## Data Availability

The datasets generated during and/or analysed during the current study are available from the corresponding author on reasonable request.
